# Concurrent pulmonary hemorrhage and deep vein thrombosis in a child with ANCA-associated vasculitis: case report and review of literature

**DOI:** 10.1186/s12969-015-0015-y

**Published:** 2015-06-10

**Authors:** Shi-Ting Tseng, Min-Hua Tseng, Jing-Long Huang

**Affiliations:** Division of Pediatric Allergy, Asthma and Rheumatology, Department of Pediatrics, Chang Gung Memorial Hospital and Chang Gung University, Linkou 5, Fu-Hsin Street, Taoyuan, Taiwan; Division of Pediatric Nephrology, Department of Pediatrics, Chang Gung Memorial Hospital and Chang Gung University, Linkou 5, Fu-Hsin Street, Taoyuan, Taiwan

**Keywords:** ANCA, Vasculitis, Pulmonary hemorrhage, Venous thromboembolism

## Abstract

Antineutrophil cytoplasmic antibody-associated vasculitis (AAV) is an uncommon but potentially life threatening disease in children. Pulmonary hemorrhage (PH) is a well recognized but lethal complication. The incidence of venous thromboembolism (VTE) is higher in patients with AAV, especially in those with active disease. However, the simultaneous occurrence of both PH and VTE has rarely been reported.

Herein, we describe a 14-year-old female with AAV who developed concomitant deep vein thrombosis (DVT) and PH within 3 days after hospitalization. She was successfully treated with timely plasmapheresis and methylprednisolone pulse therapy. VTE did not occur during discontinuation of anticoagulant. On reviewing the English literature, 5 AAV patients with coexisting VTE and PH have been reported. When faced with PH, whether or not to keep anti-coagulation treatment is a dilemma. Some of the patients kept receiving anti-coagulation treatment, whereas others undergoing inferior vena cava filter implantation. Glucocorticoids and cyclophosphamide or other immunosuppressant agents were prescribed in all patients. All of the cases survived after treatment for concurrent VTE and PH, and received short- or long-term anticoagulation treatment after discharge.

To the best of our knowledge, this is the first report of a pediatric patient with AAV presenting with coexistent VTE and PH. VTE should be considered to be a sign of disease flare-up, and early plasmapheresis with immunosuppressant therapy can rescue this fatal complication.

## Background

Antineutrophil cytoplasmic antibody-associated vasculitis (AAV) is rare but severe disease in children. It is a multi-system disease that involves the lung, skin, kidney, ears, nose, and throat. Pulmonary hemorrhage (PH) is a well recognized but lethal complication [1, 2]. The incidence rate of PH in patients with AAV is about 8-36 % [3]. The mortality rate of AAV associated PH has been reported to range from 18 % to 50 % within 1 year even under adequate treatment or mechanical ventilation [3]. Compared to PH, venous thromboembolism (VTE) is less well described. The mortality rate for AAV related VTE has been reported to be about 0–15.4 %, and most cases die of right heart failure related to fulminant pulmonary embolism (PE) [4–6]. Treatment outcome and long-term prognosis are related to early diagnosis, proper assessment of disease severity, and adequate induction remission therapy. More aggressive and timely treatment is essential especially facing life-threatening complications. Both VTE and PH have a higher incidence with increasing disease activity [4], but reports of the coexistence in patients with AAV are very rare. Herein, we present the first reported case of childhood-onset AAV complicated with deep vein thrombosis (DVT) and concurrent PH. We describe the challenges in therapeutic management for these coexisting complications.

## Case presentation

**Fig. 1:**
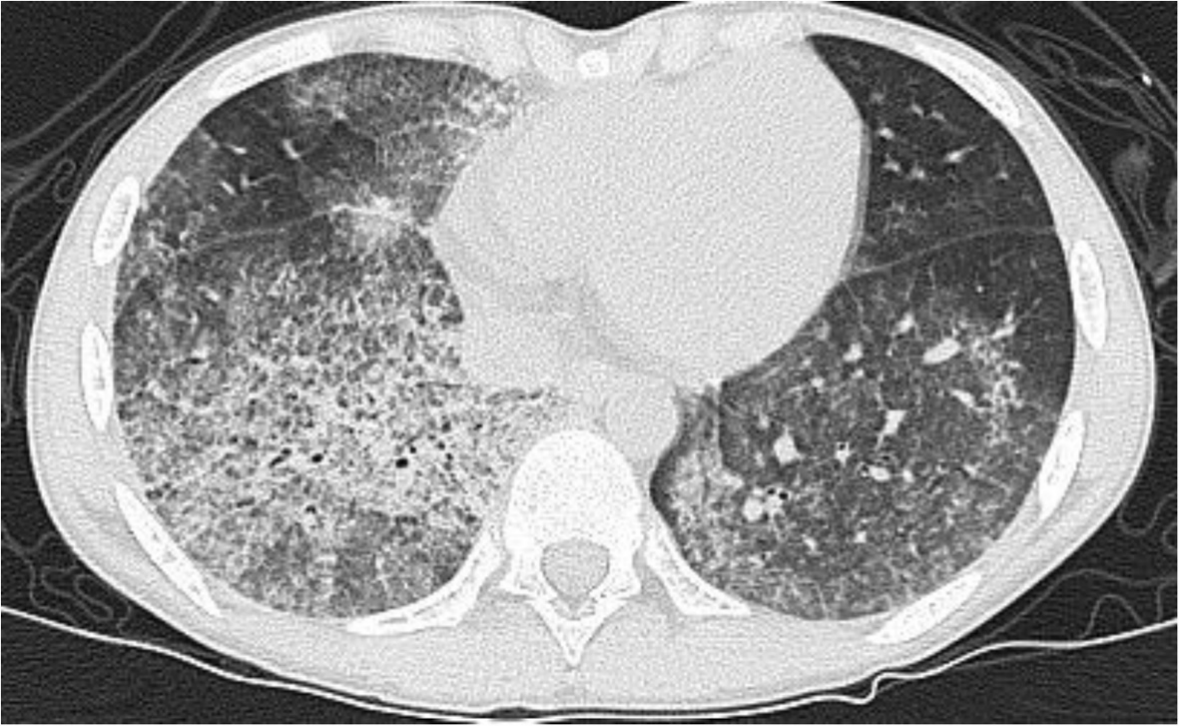
High resolution computed tomography with contrast medium showed patchy inhomogeneous opacities in bilateral lungs, more on the right side than the left side

**Fig. 2:**
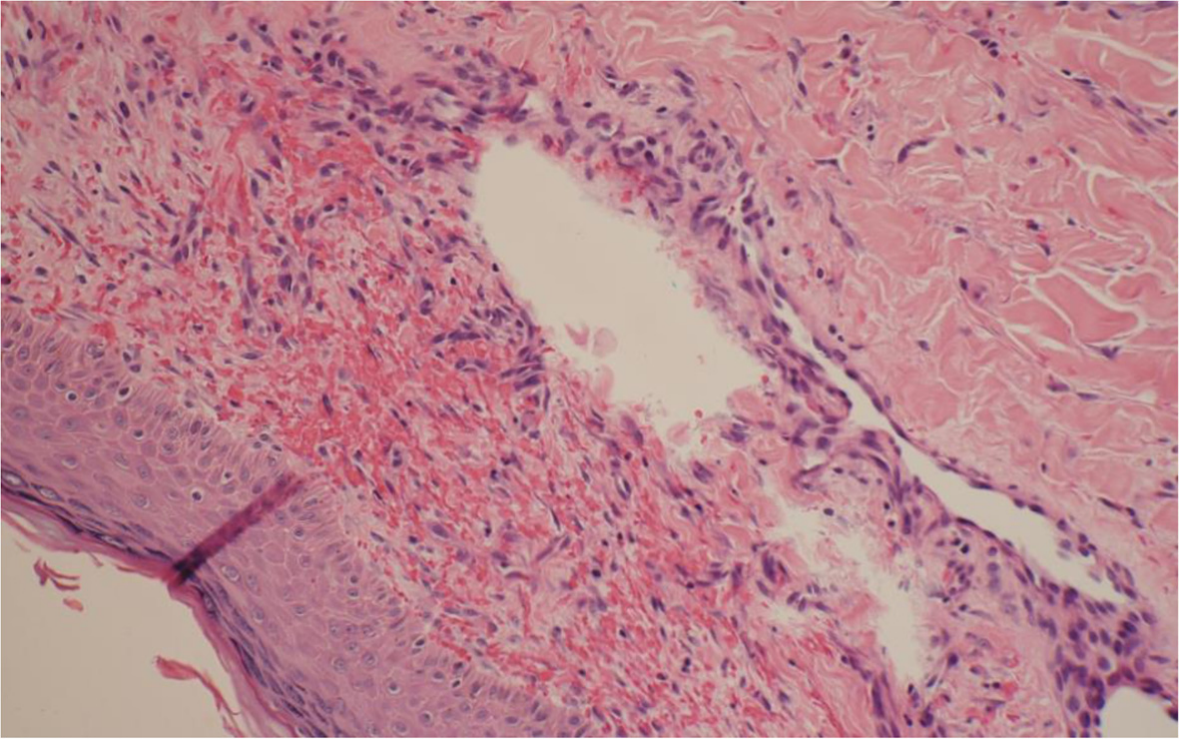
A skin biopsy revealed leukocytoclastic vasculitis in small-sized vessels in the upper dermis. Perivascular infiltrates with neutrophils, nuclear dust, and red blood cells were identified, consistent with leukocytoclastic vasculitis

This 14-year-old female was relatively healthy before this presentation without systemic diseases. She was first admitted to our hospital due to fever, hemoptysis, anemia with a hemoglobin level of 4.2 g/dL and hyperpigmented macula on her legs. High resolution computed tomography revealed PH, as shown in Fig. 1. Her clinical symptoms were response well to initial management including empiric antibiotics and blood transfusion, and no hemoptysis, anemia and fever were noted after the third hospital day. Serial examinations to survey the etiology of PH, including ruling out infections such as tuberculosis and viruses, vasculitis and other autoimmune diseases, coagulopathy and cardiac-related conditions were performed. On the sixth hospital day, serum anti-myeloperoxidase antibody (MPO-ANCA) results were found to be positive. The diagnosis of ANCA-associated vasculitis was established by the findings of a skin biopsy which showed leukocytoclastic vasculitis with negative immunofluorescence staining (Fig. 2). Renal biopsy was not performed because of normal serum creatinine level and mild proteinuria. Due to her family’s concerns of the side effects of cyclophosphamide such as gonadal suppression and infertility, we administered oral prednisolone (1 mg/kg/day) and oral mycophenolate mofetil (250 mg twice daily) to induce remission. At the end of the first hospitalization, chest radiograph revealed bilateral clear lung fields without patchy infiltration. She was discharged after 12 days of hospitalization.

Five days after discharge, she was admitted to our hospital again due to 2-day-history of swollen right lower limb. There were no fever, hemoptysis, cough, pleuritic chest pain, cyanosis or dyspnea, however skin rash was noted on her right leg. Her blood pressure was 112/85 mmHg with a heart rate of 79/min with a regular rhythm and her respiratory rate was 18 per minute with a body temperature of 37.1 °C on admission. On physical examination, swelling with different calf diameters, warmth, erythema, and positive Homan’s sign of right lower extremity, as well as multiple purpura over her right leg with local tenderness were noted. Chest, abdominal, and neurological examinations revealed no abnormal findings. The initial chest radiograph was normal without patch infiltrates, effusion, hemidiaphragm, Hampton hump or Westermark sign. The laboratory findings showed hemoglobin level of 9.1 g/dL, platelet count of 390,000/uL, white blood cell count of 12,800/mm3, international normalized ratio of 0.9, and D-dimer level of 2048 FEU ng/mL, blood urea nitrogen 30.6 mg/dL, creatinine 0.76 mg/dL, C-reactive protein 2.41 mg/L, albumin 3.33 g/dL and mild to moderate proteinuria. A Doppler ultrasound scan of her lower limbs showed a thrombus at the right common femoral vein. There were no other classic risk factors for DVT, and investigations of hypercoagulable status including anti-cardiolipin antibody, anti-β-glycoprotein antibody, lupus anti-coagulant, anti-thrombin III, protein C, and protein S were all within normal ranges. Under the diagnosis of DVT, subcutaneous low molecular weight heparin (enoxaparin; 30 mg twice daily), in addition to intravenous methylprednisolone and mycophenolate mofetil were delivered. Due to lack of associated clinical symptoms, PE was not considered. Her palpable purpura and leg swelling improved 2 days after admission.

However, she suddenly experienced hemoptysis and a decreased hemoglobin level on the third hospital day. Laboratory studies did not show coagulopathy or thrombocytopenia, and chest radiograph demonstrated diffuse ground glass appearance mixed with multiple patch consolidation on both lung fields. Due to concerns of PH, enoxaparin was discontinued to prevent further exacerbation of bleeding. Methylprednisolone pulse therapy (30 mg/kg/day) was given immediately to control the active AAV. However, the diffuse alveolar hemorrhage exacerbated with the consequent development of hypoxemia, hemoptysis, and dyspnea. She was intubated with ventilator support due to progression of PH on the sixth hospital day. Aggressive plasmapheresis was administered to remove excessive auto-antibodies. Supportive care with frequent blood transfusions and bronchoscope to remove clots were also given. An inferior vena cava filter was not considered due to resolving DVT and the concerns of a high risk with anesthesia in her critical condition. The PH resolved within 1 week after the initiation of these interventions, and she was extubated successfully on the 13th hospital day. No further hemoptysis or cough was noted during the rest of the hospitalization.

Neither DVT nor PE recurred after withdrawing the anticoagulants. To prevent further VTE, we added warfarin on the 20th hospital day. Intravenous cyclophosphamide (500 mg/m^2^) was administered thereafter. She was discharged with oral cyclophosphamide (1.5 mg/kg/day) and oral prednisolone (1 mg/kg/day). We planned to prescribe wafarin for at least 6 months if without recurrence of VTE.

She was symptom-free after discharge without hemoptysis, cough, chest pain or swollen leg. Her consciousness was clear without any abnormal neurological signs. Warfarin was prescribed and target level of international normalized ratio between 2 to 2.5 was maintained. However, she developed a sudden coma 3 weeks after the second discharge, and brain MRI revealed diffuse subarachnoid hemorrhage which favored cerebral vasculitis. After methylprednisolone pulse therapy and rituximab, she survived but in a vegetative state after that episode. We have followed this patient for 2 more years, and no PE, VTE or other relapse has occurred.

### Literature review

We searched PubMed, Medline, and Cochrane databases for literature published between 1980 and December 2014 in English, using the keywords “ANCA-associated vasculitis”, “thromboembolism”, and “pulmonary hemorrhage”. We also scanned the references of all included article for additional studies.

Only 5 cases of coexisting PH and VTE in patients with AAV were found. Table 1 shows the general characteristics, clinical presentations, and therapeutic interventions of these 5 patients and our patient [7, 8]. Three of these cases (50 %) were female and the mean age at diagnosis of AAV with coexisting PH and VTE was 36.3 years (range, 14–61 years). Beside renal manifestations, ears, nose, and throat (67 %), central nervous system (33 %), and skin (33 %) were the most commonly involved organs. One case presented with dermatomyositis, and our case presented with palpable purpura. Among these patients, 67 % had positive PR3-ANCA autoantibody, while only 33 % had positive MP3-ANCA autoantibody.

**Table 1:** Reported Cases of coexistent pulmonary hemorrhage and venous thromboembolism in patients with AAV

No.	Author	Age/Sex	Involved organ	ANCA	Presentation	Steroid (total dose)	CYC	Other agent	PLEX (N sessions)	AC	IVC filter	Outcome
1	Sousa et al. [7]	48(F)	Renal, ENT	PR3-ANCA	Simultaneous PH and PE	Y(1g)	No	MMF	Y(5)	No	Y	Survived, Recurrent PE, Long term AC
2	Sousa et al. [7]	19(F)	Renal, ENT	PR3-ANCA	Simultaneous PH and multiple PE’s, Upper limb DVT	Y(3g)	Y	No	No	UFH	No	Survived, Short-term AC
3	Sousa et al. [7]	61(M)	Renal, ENT, CNS, Skin	MPO-ANCA	PH 2 weeks after multiple PE’s	Y(3g)	No	Rituximab, IVIG	Y(5)	LMWH	Y	Survived, Pulmonary fibrios, Short term AC
4	Sousa et al. [7]	45(M)	Renal, ENT	PR3-ANCA	PH 1 week before multiple PE’s and Lower limb DVT	Y(3g)	Y	No	No	LMWH	Y	Survived, CKD stage 3, Long term AC
5	Dreyer et al. [8]	31(M)	Renal	PR3-ANCA	PH 1 week after Lower limb DVT and PE	Y(1.5g)	Y	No	Y(5)	UFH	Y	Survived, Short-term AC
6	Our case	14(F)	Renal, Skin	MPO-ANCA	PH 5 days after right lower limbs DVT	Y(3g)	Y	MMF	Y(5)	LMWH	N	Survived, Short-term AC

*M*, male; *F*, female; *Y*, yes; *ENT*, ear, nose and throat; *CNS*, central nervous system; *PE*, pulmonary embolism; *PH*, pulmonary hemorrhage; *DVT*, deep vein thrombosis; *CYC*, cyclophosphamide; *MMF*, mycophenolate mofetil; *IVIG*, intravenous immunoglobulin; *PLEX*, plasmapheresis; *UFH*, unfractioned heparin; *LMWH*, low molecular weight heparin; *AC*, anticoagulant; *IVC* filter, inferior vena cava filter; *CKD*, chronic kidney disease

Most patients with VTE had both PE and DVT (3 of 6, 50 %). Two patients had only PE, and our case had only DVT. With regards to the time sequence, 3 of these 6 patients had VTE prior to the episode of PH with an interval of 5 to 14 days. Simultaneous episodes of PH and VTE were noted in 2 patients. Only 1 patient had PH before VTE.

With regards to the therapeutic management of AAV, all patients received glucocorticoids, and 4 patients (67 %) received additional cyclophosphamide. Other immunosuppressant agents were prescribed instead of cyclophosphamide in 2 cases; 1 received mycophenolate mofetil, and the other who had the involvement of 5 organs received rituximab plus IVIG. Plasmapheresis was performed in 4 patients (67 %) after diagnosis of PH. When facing VTE with concurrent PH, the therapeutic management and considerations in these patients were all different. There were 2 cases with simultaneous PH and VTE, of whom 1 received only an inferior vena cava filter instead of anticoagulant, and the other took unfractionated heparin without an inferior vena cava filter. Three patients had VTE prior to PH, and they all received anticoagulants after a diagnosis of VTE. When PH was noted, 1 case received low-molecular weight heparin continuously, and also insertion of an inferior vena cava filter. Another case received an inferior vena cava filter first, followed by anticoagulant. In our case, we discontinued the anticoagulants when PH was noted with no implantation of an inferior vena cava filter, and began prophylactic anticoagulant treatment thereafter.

Even though three of these patients were admitted to intensive care unit for mechanical ventilation and other supportive care, all of the 6 patients survived after treatment. Four of the 6 patients received short-term anticoagulation treatment to prevent further VTE, and the other 2 patients received long-term anticoagulant treatment.

## Discussion

AAV is uncommon in childhood with an annual incidence of 0.24 per 100,000 children [2]. The incidence of PH in patients with AAV has been reported to range between 8 % and 36 % [3], while the incidence of VTE associated with AAV was less common [4]. Timely diagnosis and aggressive treatment for AAV is essential, especially when facing a life-threatening complication such as PH. There are rare reports of PH and VTE occurring simultaneously. Our patient is the first reported case of childhood-onset AAV complicated with PH and concurrent DVT. She was successfully treated with timely aggressive therapy with plasmapheresis and methylprednisolone pulse therapy, and both PH and VTE improved after the intervention.

The incidence of VTE increases during active AAV. A prospective study by Merkel et al. showed the incidence of VTE in patients with Wegener’s granulomatosis was 7.0/100 person-years compared to an incidence of 0.3/100 person-years in the general [9]. In another retrospective study, Stassen et al. found that the incidence of VTE associated with AAV was 1.8/100 person-years. During active disease, defined as 3 months before and after the diagnosis or relapse of AAV, the incidence increased to 6.7/100 person-years. A total of 198 patients aged from 14 to 81 years were analyzed in their study. Of the 23 patients (12 %) with AAV, 17 had DVT, 3 had PE, and 5 had both DVT and PE [4]. Previous studies have mainly focused on adult patients with AAV, and the same findings have also been noted in pediatric patients. One retrospective study in 2007 included 25 pediatric patients with Wegener’s granulomatosis, of whom 4 patients developed VTE during follow-up, 3 with DVT and PE, and 1 with only DVT [10]. The pathogenesis of AAV associated VTE remains unclear. Neither classic risk factors for thromboembolic events such as immobilization, trauma, malignancy, major surgery, pregnancy, positive family history nor thrombophilia show a significant prevalence in patients with AAV [6]. Several mechanisms for VTE with AAV have been proposed in previous studies. ANCAs stimulate neutrophil-induced cytotoxicity toward endothelial cells, leading to vessel wall inflammation, obliteration and damage. Other synergistic effects of infection, genetics, and drugs (e.g. hydralazine, antibiotics, non-steroidal drugs, cyclosporine, anti-thyroid agents) can trigger AAV with diverse pathological roles [11–13]. Moreover, complementary PR3 (cPR3) can also be found in patients with AAV, especially in those with PR3-ANCA. cPR3 targets plasminogen, and can delay the conversion of plasminogen to plasmin. With an increasing dissolution time of fibrin clots, cPR3 can result in a higher incidence of VTE [14]. These mechanisms can explain the higher risk of VTE in patients with active AAV, and we suggest that VTE can also be an important clue for higher AAV disease activity.

When treating VTE in patient with AAV, both timely immunosuppressive agents and anti-coagulants are important to achieve favorable outcomes. Strategies including unfractionated heparin, low molecular weight heparin, inferior vena cava filter, oral warfarin, and aspirin have been reported when facing VTE in active AAV patients [5, 7, 8, 15]. In many clinical trials, the reduction in rates of VTE recurrence were similar or even better in patients receiving low molecular weight heparin than in patients taking a combination of unfractionated heparin with a vitamin K antagonist, and the bleeding rate was not statistically different between these two groups [16]. The short-term use of anti-coagulations is usually about 6 months, while long-term use is often indicated for patients with recurrent or severe VTE, or with other acquired or genetic risk factors for hypercoagulability.

The most appropriate intervention for patients with AAV complicated with PH and VTE is a clinical dilemma, and whether or not to stop anticoagulant therapy is a difficult decision. The benefits of anti-coagulation treatment need to be balanced carefully against the potential risk of bleeding. The use of anti-coagulation therapy may increase the risk of PH, however the incidence is currently unknown due to the small number of cases of AAV with VTE and concurrent PH [7]. In previous studies, anti-coagulation treatment was usually continued to prevent further VTE even with active PH [7, 8]. In our case, we decided to discontinue low molecular weight heparin due to the active PH, and no further exacerbations of VTE were found. Plasmapheresis has been demonstrated to be beneficial in the presence of severe PH by removing ANCAs and other circulatory factors such as inflammatory cytokines, complement, and coagulation factors from the systemic circulation [14, 17, 18]. Hence, Plasmapheresis is a logistical and important therapeutic intervention for both PH and VTE [14, 17, 18]. We suggest that aggressive plasmapheresis may prevent further PH and VTE when these two complications occur simultaneously.

More aggressive therapy to induce remission may have curtailed the relapse of AAV in this patient. According to the European League Against Rheumatism (EULAR) recommendations for small vasculitis, a combination of corticosteroids and cyclophosphamide is the standard strategy of care for AAV when attempting to induce remission [19]. High-dose prednisolone at 1 mg/kg/day should be used for at least 1 month, and methylprednisolone pulse therapy may also be used if a rapid effect is needed [19]. Due to the family’s concerns of the side effects of cyclophosphamide in our case, we chose mycophenolate mofetil to induce remission. Mycophenolate mofetil can be used to induce remission, and it has been shown to achieve high complete remission rates in previous clinical trials [20–22]. We also chose prednisolone at 1 mg/kg/day, and did not administer methylprednisolone pulse therapy due to her relatively stable condition. More aggressive therapy such as methylprednisolone pulse therapy and cyclophosphamide may have led to better disease control and fewer relapses. However, we also needed to face the side effects of aggressive medication, and that decision was more difficult and less convincing to the parents as the patient was nearly symptom free at the end of the first admission.

The assessment of disease activity and predict the risk of relapse in childhood AAV remains inconclusive. In our case, we tested for MPO-ANCA several times, and the level was not found to be correlated with clinical disease severity. According to previous studies, MPO-ANCA is much more prevalent in Chinese and Japanese patients than in Caucasian patients with AAV [23, 24]. However, compared to PR3-ANCA, which was independently associated with a higher risk of relapse, the level of MPO-ANCA did not seem to correlate with disease activity [25]. In adult patients with AAV, the Birmingham vasculitis activity score (BVAS) has been validated as a comprehensive multisystem disease activity assessment tool. A retrospective study published by Morishita et al. assessed the performance of the BVAS for the diagnosis of children with AAV. The median BVAS v.3 at the time of diagnosis (18.0 + − 8.0) was similar to the mean BVAS v.3 reported in adult patients with vasculitis, however the pediatric AAV patients had higher scores for pulmonary and renal involvement. Before the BVAS v.3 can be used as a disease activity assessment tool in pediatric AAV, further modifications in weighting or changes to the ceiling scores may be required [26]. In addition, Walsh et al. assessed 535 patients for the risk factors of a relapse in AAV, and found that anti-PR3 positivity and cardiovascular involvement were independently associated with the first relapse, whereas neither BVAS score nor C-reactive protein level showed a correlation with the first relapse [27]. Moreover, pulmonary-renal syndrome is defined as diffuse alveolar hemorrhage and rapidly progressive glomerulonephritis. Therefore, regularly monitoring hemoglobin level, hematuria, proteinuria, and serum creatinine is important for further assessments [28, 29]. Other studies have reported that patients with AAV have a higher incidence of VTE, especially in the active stage [4, 5, 7, 15], however VTE is not included in scoring disease activity in the current BVAS. We suggest that VTE is a clue of active AAV and should be included in further scoring systems to more accurately assess disease activity. Overall, we suggest that serum creatinine level, BVAS, falling hemoglobin level, and episodes of VTE should all be taken into consideration when assessing the disease activity of children with AAV.

The prophylactic anticoagulant therapy in patients with AAV is in still under debate. Patients with AAV have a higher incidence VTE. However, patients may also suffer from PH or cerebral hemorrhage more frequently during the active stage. Prophylactic anti-coagulation may therefore not be safe or appropriate for every patient. If the patient has recurrent or refractory thromboembolism, adequate prophylactic anti-coagulation treatment may be helpful. Whether or not to use prophylactic anti-coagulant agents depends on the patient’s clinical condition. Further prospective studies are needed to develop reliable treatment guidelines for VTE in patients with AAV.

The strength of this report is that when facing the dilemma of concurrent PH and VTE, we used timely aggressive therapy with methylprednisolone pulse therapy and plasmapheresis and other supportive care to resolve PH and keep VTE under control. The limitations of this study are as following. First we did not perform angiography of pulmonary computed tomography or a ventilation-perfusion scan during the second hospitalization. It is therefore difficult to completely rule out concurrent PE, especially with the active PH. Second, we chose mycophenolate mofetil instead of cyclophosphamide during the first hospitalization after discussing the benefits and side effects of both medications with the parents. We do not know whether the outcome would have been better if we had chosen cyclophosphamide for the initial induction remission therapy.

## Conclusion

AAV is an uncommon and multi-systemic disease in children. The incidence of VTE is higher in patients with AAV, and VTE can be an important clue of severe active disease. When patients develop VTE, anti-coagulation therapy is indicated to treat and prevent recurrent episodes. However, the treatment intervention is challenging when facing complications of VTE and concurrent PH, which can occur in a 14-year-old girl. The administration of timely methylprednisolone pulse therapy and plasmapheresis can decrease disease activity and lead to complete remission of both VTE and PH.

## Consent

Written informed consent was obtained from the patient’s father for the publication of this report and any accompanying images.

## Abbreviations

AAV: Antineutrophil cytoplasmic antibody-associated vasculitis
PH: Pulmonary hemorrhage
VTE: Venous thromboembolism
DVT: deep vein thrombosis
PE: Pulmonary embolism
